# Social Isolation in Community-Dwelling Older Adults During COVID-19: Understanding the Role of Resilience

**DOI:** 10.1177/00469580221148880

**Published:** 2023-01-20

**Authors:** A. Garnett, K. Prentice, R. Booth, L. Donelle, J. B. Orange, F. Webster

**Affiliations:** 1 Western University, London, ON, Canada; 2 University of South Carolina, Columbia, SC, USA

**Keywords:** social isolation, resilience, COVID-19, older adults, community

## Abstract

The COVID-19 pandemic increased social isolation for many older adults, causing concern for their health and well-being. To enhance understanding of how community-dwelling older adults were impacted by prolonged social isolation during COVID-19, a qualitative descriptive study was conducted to: (1) explore the self-reported factors supporting their resilience during COVID-19 related social isolation, and (2) to help understand the intentional and unintentional outcomes of the government mandated health measures. A total of 19 community dwelling older adults were sampled. Factors that supported older adults’ resilience during COVID-19 included maintaining positivity, drawing on historical experiences of resilience and finding opportunities to connect with their community. However, collective safety came with losses: such as time, freedom, opportunity, engagement, and initiative. The findings provide insight on contributing factors to resilience against social isolation in older adults and suggest the value of collective, community-based approaches to build resilience across variable contexts in this population.

**What do we already know about this topic?**
Many older adults living in community settings experienced an extreme increase in social isolation during the COVID-19 pandemic, yet some older adults were more resilient to the impacts of social isolation than others.
**How does your research contribute to the field?**
Our research extends the knowledge base on the factors that contribute to community-dwelling older adults’ resilience to social isolation particularly in the context of limitations on in-person gathering.
**What are your research’s implications toward theory, practice, or policy?**
Implications arising from this research include: Mitigating the impacts of social isolation on community-dwelling older adults requires community informed approaches that foster new skills and embrace older adults’ previous experiences in overcoming adversity.

## Introduction

Globally, the COVID-19 pandemic has had widespread impact on many older adults causing extensive loss of life, and over time, declines in health and well-being.^1^ Health as defined by the World Health Organization “is the condition of having complete physical, mental, and social well-being, and not merely the absence of disease or infirmity.”^2^ Extensive public health measures implemented in Canada restricted physical contact and in-person gatherings which led many older adults to become socially isolated. While these protective measures were intended to mitigate the spread of disease, they also had negative health impacts on some older adults, causing disturbances in sleep, increased rates of depression, and fatigue among other effects.^3^ Social isolation, one unintended consequence of COVID-19-related government policies, is defined as the objective lack of meaningful contact and communication with contacts.^4^ Prolonged social isolation in older adults is associated with cognitive decline, physical de-conditioning, increased frailty, and increased use of health services including physician visits.^5-7^ Research on previous pandemics has documented some of the negative impacts of social isolation on the mental health of older adults. For example, during the SARS 2003 outbreak in Hong Kong, there was a spike in suicide rates in older adults.^8^ Research on the mental health effects from the H1N1 pandemic in 2009 highlighted the need to address mental health ahead of future pandemics.^9^ The COVID-19 pandemic has been globally more extensive and prolonged than any recent pandemics suggesting that the potential negative health impacts may reach unprecedented levels. Given the protracted nature of the COVID-19 pandemic it will be important to understand the longer-term impact of and contributors to resilience against social isolation in older adults.

Canadian federal and provincial government-imposed restrictions, widespread fear of contracting COVID-19, and several waves of re-infections, are all potential contributors to the unprecedented levels of social isolation experienced by some community dwelling older adults.^10^ Peng and Roth^11^ indicated that older adults who exercised precautions such as avoiding public settings, were more physically isolated than others who did not follow such precautions. Ironically, the COVID-19 pandemic and public health measures designed to protect the physical health of older adults may have produced unintended negative impacts on older adults’ mental health by causing prolonged social isolation. Emerging findings from COVID-19 pandemic research demonstrates that older adults and their caregivers have experienced very high levels of anxiety, depression, and loneliness arising from social isolation.^12,13^

At the population level, social isolation and its potential negative impacts on the health and well-being of older adults, such as loneliness, are challenging for the health system to manage.^14^ Loneliness, a distinct construct from social isolation, is the subjective feeling of being isolated.^15,16^ According to Chatters et al,^17^ living arrangements do not necessarily determine social isolation in older adults. The impact of social isolation can be tempered by social participation in activities and through individuals’ resilience.^14^ Windle^18^ defines resilience as the process of effectively negotiating, adapting to, or managing significant sources of stress or trauma. It is the capacity and willingness of the individual to use personal resources such as positive reframing and agency, meaning and purpose, and acceptance and belonging that buffers them from adversity.^19^ Community context and social policies that support older adults’ participation in collective agency, leadership, engagement, and shared decision-making help facilitate resilience.^19^ However, there is a knowledge gap with regard to what extent individual protective factors and other health and social equity attributes such as digital health literacy may impact older adults’ resilience in the context of prolonged social isolation experienced during the COVID-19 pandemic.

Increased understanding about how the multi-dimensional construct of resilience in community-dwelling older adults was impacted by COVID-related social isolation could help inform the development of community programs and initiatives to support older adults and particularly those who are most vulnerable. For example, drop-in programs held by local libraries could provide access to technology and appropriate training to enable older adults to foster new skills and to develop broader social networks using web-based tools. Moreover, knowledge pertaining to resilience among older adults who were isolated during COVID-19 will be valuable to policy makers, community agencies and family members as we continue to navigate the current pandemic and make meaningful change based on current experiences. Additionally, further attention to why some older adults are more resilient against social isolation than others (or potentially even thrive) in the context of COVID-19 could help inform future pandemic preparedness.^20^

Many researchers have pointed out that resilience might not be an individual characteristic (eg, positive outlook) but include an interaction between individual traits and parameters related to community environment and health equity such as social class, status, race.^18,21^ This perspective aligns with community resilience theory whereby resilience of the whole (community) is characterized by the mobilization and enhancement of individual and community resources thereby contributing to collective action to overcome and even thrive in an environment of uncertainty, instability and adversity.^22-24^

A body of research was conducted on older adults’ resilience during the earliest part of the COVID-19 pandemic.^3,25-27^ However, these studies largely focused on the earliest stages of the pandemic and therefore do not capture older adults’ experiences as the pandemic and concomitant government mandates varied and persisted over time. Therefore, this research sought to explore older adult’s experience of resilience in the context of social isolation over a longer duration (approximately 18 months) of the COVID-19 pandemic. The purpose of this study was: (1) to explore the self-reported factors that supported the resilience of community-dwelling older adults who experienced COVID-19 related social isolation, and (2) to understand the impacts of government-mandated COVID-19 regulations that restricted in-person engagement on the health and well-being of community-dwelling older adults. Questions guiding this research were, “What are the self-reported factors that have supported older adults’ resilience in the context of COVID-19 related social isolation?” and “What are the outcomes of government policies that have restricted in person engagement on perceived social isolation in community dwelling older adults?”

## Method

Informed by a social constructivist lens,^28^ a qualitative descriptive approach^29,30^ with content analysis^31^ was used to explore the role of resilience on perceived social isolation in older adults living in the community during the context of the COVID-19 pandemic. Ethics approval for this study was granted by [blinded for review] University’s Non-Medical Research Board.

### Setting and Context

Data collection took place between June and August 2021 during the COVID-19 pandemic in a mid-sized city in southwestern Ontario, Canada with a large older adult population.^32^ In Ontario, Canada, government-mandated safety regulations changed over the course of 2 years. Public Health safety measures were instituted in March 2020 and consisted of a lockdown, which involved the closure of all businesses.^33^ Citizens were advised to stay home and only be in contact with those within their “social bubble” of up to five people.^33^ By July 2020, some of these restrictions were lifted and some businesses opened with capacity limits.^34^ In December 2020, the number of cases of COVID-19 began sharply increasing in Ontario, leading to another province wide lockdown that severely limited in-person gathering to people within the same household.^35^ Between December 2020 and June 2021 there were two more province wide lockdowns.^33,35^

### Sampling and Participants

In order to obtain a community-based sample of older adults during COVID-19-related restrictions on social gathering, a point of contact was required. Therefore, a convenience sampling strategy was employed and a database was used to identify the target population.^36^ The database consisted of members of a university research center on aging, exercise and activity. Members registered in the database had previously consented to be contacted for research purposes and this membership registry [anonymized for confidentiality purposes] enabled us to identify a broad-based sample of community-based older adults and invite them to participate in the study. Older adults registered in the database who were 60 years of age or older, spoke English and who lived independently in a community setting met the study inclusion criteria and were invited to participate in an in-depth interview. Telephone or Zoom video conferencing was used to obtain informed consent and to conducting the in-depth interviews with study participants. Single interviews were conducted with one member of a household.

### Data Collection

Data were collected using semi-structured interviews that were informed by the available body of literature on resilience within older adults and by drawing on the social relational and community components of the community resilience theory (eg, ability to access community resources such as infrastructure, engagement with community resources)^24^ (see Table 1). The resilience literature focused on different types of resilience and were adopted and included in the interview guide, such as physical and mental,^3,37,38^ social and relational^18,39,40^ and institutional resilience and community resilience.^9,18,24,41^ These types of resilience were incorporated into the interview guide to address the different ways participants’ experiences contributed to their overall resilience. The findings from these studies focused on the actions employed by older adults to build or maintain resilience such as engaging in physical activity,^36^ maintaining social connections within their communities and drawing on support from their members of their communities^3,18,24,37,40^ maintaining daily routines and learning new skills.^3^

**Table 1.** Interview Guide Informed by Resilience Concepts.

Dimension	Question	Objectives met	Citation
Institutional	How has the pandemic impacted your life? (prompt for accessing resources, dealing with stressors, health habits). What resources have you used to manage with the stresses of the pandemic?	1	Abrams and Szefler^41^
	How has the pandemic affected your ability to access your basic needs? (eg, grocery shopping, pharmacy, doctor’s office etc.?) What strategies have you used to cope with these changes? How do you feel about this? (accessing resources)	1 and 2	Windle^18^
	How has the pandemic affected your ability to access other needs within your community? For example, attending church/mosque, social clubs, hair salon (Windle—other resources that may impact well-being)	1	Magis^24^ and Windle^18^
	Can you describe how you feel when going out in public? (safety)	1	Huremovic^9^
Social	Can you describe your interactions with others? (who do they know, who are they in contact with/what kind of relationships?)	2	Netuveli et al^39^
	Can you describe how connected are you to these individuals/groups? Have you used community resources to achieve this? (listen for if they can count on them for comfort)	2	Magis^24^ and Netuveli et al^39^
	Can you describe how you have maintained contact with your social circles? (what resources have they used to stay in contact?) What has made it difficult to stay in contact? (Challenges using technology?)	1 & 2	Tsai et al^40^
	Can you describe how have you coped with letting go of social circles within your community? (being away from others affecting mental health)	2	Magis^24^ and Netuveli et al^39^
	What resources have you accessed in the community to stay connected? (neighborhood resources)	1	Magis^24^ and Windle^18^
	What significant events have happened to you or your family/friends over the past year? How have they made an impact on your life? (coping with adversity)	2	Magis^24^ and Netuveli et al^39^
Physical/mental	How has the pandemic situation affected your physical health? Mental health? How have you adapted to your new situation? (maybe prompt for how its affected their quality of life?)	2	Harden et al^3^ and Whitson et al^38^
	What do you do for exercise? How frequently do you exercise? How has your activity been impacted since the pandemic began? What have you changed? How does participating in activity make you feel? (Do health interventions optimize physical resilience?)	2	MacLeod et al^37^ and Whitson et al^38^
	Are there any other activities you engaged in, or places you accessed/attended prior to the pandemic that you were no longer able to participate in as a result of the pandemic? (community resources, proximity, social support) Were there alternative strategies you could use to continue to engage with these services?	2	Magis^24^ and MacLeod et al^37^

The interview questions explored: (1) if and how they experienced social isolation during COVID-19, and what self-reported factors and community-level factors supported their resilience during this time, and (2) how the participants experienced mandated restrictions that limited their ability to participate in in-person social gathering with family and community members. Duration of interviews ranged from 30 min to 1 h and were conducted using Zoom video conferencing technology (n = 11 participants) or telephone (n = 8). All interviews were audio-recorded and transcribed verbatim to enable in-depth analysis. To help ensure study rigor, an audit trail was used to keep track of participant details such as timelines of when they were contacted and their method of engaging in the interview and notes about the participant, including demographic information.^42^ Additionally, reflexive notes were recorded after each interview to enhance the rigor of the study.^42^ Throughout the iterative and concurrent process of data collection and analysis, two of the study researchers (AG and KP) engaged in ongoing discussions to ensure that the interview questions were eliciting a rich description of the phenomenon of interest.^42^

### Data Analysis

The construct of resilience informed by the extant literature and the community resilience theory was organized into the following dimensions: institutional, social, physical, and mental as a means of informing the development of the interview guide and in order to help situate the findings from the current study. In accordance with qualitative content analysis^31^ the data was systematically coded in conjunction with recording insights and reflections on the data. The first and second author co-coded the data (AG and KP) to ensure consistency.^42^ Additionally, the entire research team was consulted on the preliminary coding and at consistent intervals throughout the analytic process.

The coded data was then examined and sorted to identify similar patterns or themes. The authors also searched for commonalities and differences within the data and decided on generalizations that held true for the data, and examined generalizations compared to existing knowledge.^43^

## Findings

A sample of 19 older adults, including 15 women, with a mean age of 74.8 years and age range of 64-90 years were recruited to participate in the study (Table 2).

**Table 2.** Demographic Table.

Age	Gender	Marital status	Education	Religion	Are you actively practicing?	Household income	Type of home	How many people live in your home?	Main form of transportation
74	Female	Married	Bachelor’s degree	Christian	No	$60,000-$80,000	Two story house	1	By city bus
80	Female	Divorced/separated	Bachelor’s degree	Unitarian	Yes	Below $20,000	Apartment	1	By city bus
86	Male	Married	Bachelor’s degree	Atheist	No	$60,000-$80,000	Two story house	2	By car, I drive myself
90	Male	Married	Graduate degree	Christian	No	$40,000-$60,000	Two story house	2	By car, I drive myself
78	Male	Married	College diploma	Christian	No	$100,000+	Bungalow	2	By car, I drive myself
73	Female	Divorced/separated	Graduate degree	Christian	Yes	$60,000-$80,000	Townhouse	1	By car, I drive myself
70	Female	Married	Bachelor’s degree	Christian	No	$100,000+	Cottage (2 levels)	2	By car, I drive myself
73	Female	Married	Graduate degree	Jewish	Yes	I prefer not to say	Two story house	2	By car, I drive myself
70	Female	Single	Bachelor’s degree	Christian	No	$80,000-$100,000	Condo	1	By car, I drive myself
75	Male	Married	Bachelor’s degree	Christian	Yes	$40,000-$60,000	Split	2	By car, I drive myself
64	Female	Divorced/separated	Bachelor’s degree	I prefer not to say	No	$40,000-$60,000	Condo	1	By car, I drive myself
72	Female	Married	Bachelor’s degree	Jewish	Yes	I prefer not to say	Condo	2	By car, I drive myself
74	Female	Married	Graduate degree	None	I prefer not to say	$80,000-$100,000	Single floor attached condo	2	By car, I drive myself
72	Female	Widowed	Bachelor’s degree	Christian	No	$100,000+	Condo	1	By car, I drive myself
73	Female	Divorced/separated	Bachelor’s degree	Pagan	Yes	$20,000-$40,000	Apartment	1	By car, I drive myself
71	Female	Married	Bachelor’s degree	None	No	$100,000+	Bungalow	2	By car, I drive myself
71	Female	Other (specify)	College diploma	Christian	No	$20,000-$40,000	Apartment	1	By car, I drive myself
69	Female	Married	High School diploma	Christian	Yes	$60,000-$80,000	Two story house	2	By car, I drive myself
87	Female	Widowed	Bachelor’s degree	Christian	No	$20,000-$40,000	Bungalow	1	By car, I drive myself

Two overarching themes were identified from the analysis: *adapting to new contexts* and *government actions fostered safety at a cost*. Theme 1 focused on how participants demonstrated resilience during the pandemic through maintaining positivity, taking the opportunity to develop new skills, channeling new strengths, and continuing to interact with the community despite constraints on in-person gathering. Theme 1 was categorized into four subthemes a) clear intentions to maintain positivity, b) “gifted with time,” c) finding strength through isolation, and d) understanding the importance of community. Theme 2 focused on how government actions fostered safety but that this came at a cost to participants’ emotional health, which negatively impacted their ability to remain resilient. Theme 2, *Government actions fostered safety, but came at a cost*, was categorized into three subthemes a) supporting the collective through individual compliance, b) mourning loss of freedoms, and c) experiencing tensions from living in chronic fear (Table 3).

**Table 3.** Resultant Themes and Sub-Themes.

Main theme	Sub-themes
Adapting to new contexts	(a) Clear intentions to maintain positivity
	(b) “Gifted with time”
	(c) Finding strength through isolation
	(d) Understanding the importance of community
Government actions fostered safety, but came at a cost	(a) Supporting the collective through individual compliance
	(b) Mourning loss of freedoms
	(c) Experiencing tensions from living in chronic fear

### Theme 1: Adapting to New Contexts

In this overarching theme participants describe how they intentionally took steps to maintain positivity in their life during the COVID-19 pandemic. Participants viewed the pandemic as an opportunity to develop new or rekindle previous skills and how they used their experience of isolation to channel new or rejuvenated strengths. Further, participants discussed how they understood the importance of community by continuing to be involved in different ways throughout the pandemic.

#### Sub-theme 1-1: Conscious intentions to maintain positivity

In this theme, participants describe how they worked to maintain their well-being during the COVID-19 pandemic. The participants discussed their belief that stepping away from engaging in COVID-19 matters assisted them in maintaining resilience. One participant disengaged from COVID-19 by acknowledging her lack of control over the situation, “. . . it’s just really disengaging from it . . . media, being television, being radio, whatever . . . you need to get away from that. Not to hide your head from it, but just step away from it. I don't control it all . . .” (P3). This participant reflects the general sentiment among participants in how choosing to step away from media helped her maintain her mental well-being during the pandemic. The participants indicated that by engaging in exercise, they could maintain their physical well-being in order to build resilience against COVID-19. Some participants described going for walks as helpful for keeping busy or staying in shape when they were not allowed to leave the house. One participant indicated that her and her husband knew to prioritize physical activity in order to maintain their physical and mental well-being during the lockdowns,

The first thing . . . we [decided was] that exercise [and] mental health was going to be important. And . . . my husband has a stationary bike . . . he would use . . . Whereas . . . I would go out for a walk right after breakfast. And that was done very deliberately in order to get the endorphins up in order to . . . start the day on the right foot. (P10)

This participant highlights how exercise supported their resilience on a daily basis during the pandemic. This was demonstrated by another participant who indicated how being at home gave her the chance to develop a healthy routine, something that had been missing from her life,

But since the pandemic . . . I’m at home all the time. And so that has meant that life has settled into a very regular pattern, which I think probably is healthier . . . I get up and [attend] mass . . . every morning and that just anchors my day . . . it both gives structure to the day but also has been a chance for . . . [a] real deepening of my spiritual life. (P13)

This deepened spiritual connection was described by P13 as assisting in her maintain mental wellness as well as assisting her in building resilience. The participants also indicated maintaining their mental well-being was essential to sustaining resilience during COVID-19.

However, there were situations where the older adults struggled to adapt to the social distancing regulations established due to the pandemic. One participant described how the pandemic affected her mental health and what she did to cope with the emotions she was experiencing:

Respondent: Oh, I became . . . borderline depressed . . . almost from the beginning.

Interviewer: How have you coped with that?

Respondent: Badly. I cocoon myself even more and you know, if I’m making a trip to go and get some scones . . . I end up walking out with a hell of a lot more than scones. And coming home and scarfing them all, I have medicated myself with bad choices and food . . . I’m feeling as heavy as I am, it’s not imaginary it’s real. I have less energy; I have less desire to do things. I’m slightly short of breath on exertion now . . . it has not been pretty. (P8)

In this way, the participant found the COVID-19 pandemic challenged her capacity to be resilient. Furthermore, having to adapt to the social distancing regulations in innovative ways worked for some, but not others. One participant suggested she did not enjoy the garage visits that were meant to replace in-person gatherings, “I just don’t want to do that anymore, it was horrible . . . I mean it was nice to be with our friends but the whole thing of sitting in somebody’s garage is just kind of beyond me . . . it’s affected our social life” (P6). This participant revealed that she felt frustrated that her social life had been impacted by these social distancing restrictions.

#### Sub-theme 1-2: “Gifted with time.”

Sub-theme 1-2 describes how the participants highlighted positive elements that came with the pandemic, such as the gift of time to engage in activities they might not have done otherwise, helping them to build resilience from social isolation. For instance, participants suggested the pandemic was an opportunity to learn and engage in new activities. One participant described how the pandemic allowed her time to try new recipes, “I used to never bake at all . . . I tried a whole lot of new things that I’ve never done before, so my food repertoire’s vastly increased” (P5). Having time allotted to try baking new things helped this participant to adapt to her changing circumstances. She was able to use her time in productive ways, supporting her resilience. Another participant took the time to focus on learning Spanish when the lockdown began, “I started Spanish about three years ago but I really started to [study] seriously [when] COVID began. I had more time” (P1). This participant acknowledged that he had been gifted with time to pursue his interest in learning a new language. Other participants either took on new interests or rekindled existing ones, such as P10 who reacquainted herself with yoga practice, “. . . I would take those books and read them . . . become acquainted with everything . . . it just deepened what I was doing because I learned a bit more . . . Or in the case of yoga I relearned some things that I’d kind of forgotten . . .” (P10). The lockdowns provided some participants with the time to reengage with past activities they used to enjoy, and even helped improve skills.

#### Sub-theme 1-3: Finding strength through isolation

This sub-theme discusses how participants sought inner strength to build resilience during the COVID-19 pandemic. Some participants had built resilience in their past and believed this helped them to be resilient during the pandemic, and others built their resilience because of the pandemic. For example, one participant described leading an isolated way of living and self-reliance from a young age, “. . . because of my experience of isolation during . . . my childhood . . . not [having] family or friends I learned to rely on myself. Independence is of utmost importance for me . . . I still drive and do my own shopping, cooking and light housekeeping” (P7). She explained how becoming resilient as an adult, eased her experience of social isolation during the pandemic. COVID-19 provided opportunities to enhance resilience; one participant stated he learned how to do online banking, “. . . I [learned] to rely on myself. Sometimes it’s frustrating, sometimes it’s satisfying to master a bit of technology” (P1). Further, he alluded to how relying on his own abilities to access services helped him to adapt during the pandemic. Similarly, others relied on themselves for certain services, such as cutting their own hair because they could not access the hair salon. They would use their own resources, such as purchasing barber scissors or razors. Some participants also actively sought activities to keep them busy to adapt to their new situation, such as working on projects, “I’ve just been concerned about keeping busy, I was working on this project, but I’m always just trying to put one foot in front of the other, just [to] keep going” (P9). By creating projects for themselves, participants felt that they supported their own resilience. The participants also described an attitude of acceptance. One participant suggested she had to accept the restrictions and be creative about ways to adapt,

. . . you have to be creative sometimes . . . I’ve taken to online purchases when you can’t go into a mall and get what you need . . . instead of going to the gym I’ll do the walking or the exercise in the house. I think that’s probably the only resources that I’ve needed . . . to adapt to . . . it’s all very possible and doable, it’s just different. (P11)

This participant revealed that a frame of mind could be impactful when deciding how they wanted to adapt to regulations where they had no control.

#### Sub-theme 1-4: Understanding the importance of community

The participants underscored the importance of community to building resilience and remaining connected to others by continuing to volunteer and engage with the community. One participant described the ways she stayed involved prior to the COVID-19 lockdown, “. . . I volunteer at the Food Bank. I play at the [name] Bridge Club. I go to the gym. I’m in a book club. I look after my grandchildren. We like to travel and, you know . . . I’m used to being busy . . .” (P16). She also mentioned that some of these in-person events switched to online platforms during the pandemic and she had to learn to use technology to stay connected, “I’ve been playing bridge online and . . . I learned how to use Zoom . . . And then instead of going to the gym I started walking around the block and walking through the local little parks and that kind of thing” (P16). This participant revealed how she took the initiative to learn how to sustain her usual activities through the use of online programs to adapt to the social distancing regulations. Other participants connected to friends and family through technology too, however, some did not learn new technology. One participant explained that she had stopped using technology once she retired, which made the return challenging,

. . . I am not great [with] technology. It’s the old theory, if you don’t use it you lose it. [Before] I retired . . . I was so technical . . . we had everything. Blackberries . . . tablets . . . I used all that technology. Once I retired, I didn’t use it anymore. I’m very uncomfortable with the computer . . . I use it because I have to. I’m frustrated with a society that’s putting everything online because I have great difficulty with that now . . . (P14)

This participant reveals her frustration with what she perceives as the forced need to be familiar with technology to access basic services.

### Theme 2: Government Actions Fostered Safety at a Cost

#### Sub-theme 2-1: Supporting the collective through individual compliance

In this theme we discuss how the participants understood the importance of following the government mandated restrictions to inhibit the spread of COVID-19 among themselves and others they cared for. Many took action to support public health mandates by wearing masking, staying their distance from others and getting vaccinated.

One participant expressed her view that it was common sense to follow protocol, “you . . . [wear a] mask, you protect yourself, that’s the smart thing to do, just do it” (P8). They believed that if they took these actions to protect themselves and others, they would be supporting the province on its way to recovery. Another participant described how they were working to protect others, “We are super conscientious, and have been . . . about following the rules, so we haven’t seen our friends. We had nobody come to our house; we haven’t gone to anybody’s house” (P6).

Some participants felt that getting vaccine was another way they could help prevent the spread of COVID-19 in the province. One participant described how the vaccine gave her hope that she could spend more time with others, “. . . now that I have two vaccines, I feel pretty good. I know that there’s still a chance that you can get it, but it’s not going to take your life . . . I feel a lot safer. I feel relieved” (P12). This participant was able to feel some comfort that she could still be safe and social amid the social distancing regulations. Some participants discussed other ways they were social with others while continuing to follow the government-mandated regulations. For example, one participant explained how they negotiated the rules with their neighbours by continuing to keep in contact, “. . . none of us have actually got together, but we’ve . . . spoken at a distance with masks on, either across the backyard fence or out on the driveway . . . everyone’s still being sociable . . .” (P15). In this way, the participant could remain safe and comply with government mandates while continuing to meet their social and emotional needs.

#### Sub-theme 2-2: Mourning loss of freedoms

This theme highlights the participants’ expressed emotional distress that they experienced over loss of freedoms over the course of the pandemic. The participants experienced fear, sadness, frustration, and confusion over loss of seeing others, attending significant events, and traveling. For some participants, the lockdowns were an inconvenience but for others it caused emotional turmoil. For example, one participant had moved to the city 3 years ago and explained she had already felt isolated, but the pandemic worsened things when she could not do what she wanted to do:

Oh, [it] absolutely isolated me. It—I couldn’t move around freely, I had to come back from Florida early and I just felt like I was in a prison . . . Even though I would go out to the grocery store [and] see my grandchildren, I just felt imprisoned. (P8)

Her expression of feeling like a prisoner highlights the magnitude of the impact the government restrictions had on this participant’s sense of autonomy and perceived ability to engage with others within her social circle. Another participant suggested she stopped engaging in self-care:

[You see] how much your priorities really change, how stuff didn’t matter so much, going out shopping for clothes didn’t matter anymore, you had so much clothes in your closet, just wore the same thing over and over . . . Well who cares, who’s going to see you? (P18)

While this participant noted a change in her priorities that came with COVID-19, she also expressed dejectedness brought on by ongoing isolation which included the mundaneness of reliving the same day without interaction with someone else.

### Sub-theme 2-3: Experiencing Tensions From Living in Chronic Fear

The participants expressed ways they experienced tension and fear during the pandemic, including while in lockdown and interacting with others in public. For some, being close to others made them nervous due to the possibility of catching COVID. One participant indicated she was always alert of who was near her, particularly in the grocery store,

I'm very aware of how close I am to anyone . . . whether they’re wearing their mask properly, and how close to get to them if they're not wearing their mask properly, coughing or sneezing . . . I’m very aware of what you touch and groceries. (P19)

The effects of isolation caused some participants to behave differently than normal, to be conscientious of their actions and the actions of others. For some, fear was developed at home during isolation. One participant discussed how the media cultivated fear,

I remember in the beginning I would write down the case [count] every day . . . I was . . . focused on it, because it was terrifying . . . I watch the News every night . . . I [also do] online research . . . like CBC, I . . . trust them. I [wanted to] see how we’re doing in Canada . . . in [current city], compared to, you know England or wherever . . . to keep things in perspective. (P17)

Writing down the numbers each day was this participant’s way of coping with the uncertainty of the pandemic and relied on media outlets to “keep things in perspective.”

## Discussion

The results of this study include a rich description by community-dwelling older adults of some of the self-reported factors that impacted their resilience during COVID-19 related social isolation. Key findings include: older adult participants adjusted their frame of mind and took conscious actions (such as learning to use technology to remain socially connected) to help them maintain their resilience throughout COVID-19; drawing upon skills learned over their lives to helped them cope; and government mandates were accepted as necessary by most but for some they led to a profound sense of loss and isolation that was not easily overcome.

Situating our findings within a formalized definition of resilience as the process of effectively negotiating, adapting to, or managing significant sources of stress or trauma,^18^ we found that many older adult participants exhibited characteristics that would suggest they were resilient. These older adults described different strategies that enabled them to maintain optimism throughout the COVID-19 pandemic, including actively disengaging from negative influences such as the media, viewing the lockdowns as “gifted with time” and drawing on inner strength gained through past experiences. Corroborating early COVID-19 research that determined that strategies such as mindfulness and optimism could be protective against negative mental health effects^44,45^ our results extend the literature by suggesting that these protective measures may continue to be effective over longer periods of time.

Interpreting the study results within the framework of community resilience, the findings suggest that staying connected to members of their community was a key strategy for adapting to the mandates which required physical distancing. MacLeod et al^37^ found that older adults who continued to remain involved with their communities as they aged demonstrated higher resilience than others. Additionally, Netuveli et al^39^ indicated that individuals who were more resilient had a higher prevalence of social support than those with low resilience. The participants in the current study remained engaged with their community through their continued involvement with previous volunteer commitments or social clubs facilitated by learning to use technology for the purposes of social engagement. In doing so, their efforts contributed to their personal and community resilience, highlighting the relational nature of social engagement and suggesting that individual resilience depends on more than individual level factors.

Notable in the current study was the initiative taken by participants to use the time during the lock down measures to develop new skills or reconnect with previous skills they had learned in the past. These actions served to empower them and fostered their sense of resilience in the context of adversity. Some study participants described how their prior experience with adversity helped them cope with the COVID-19 pandemic and suggests that resilience has a strong experiential component that can be transferrable across many parameters such as time and context. This finding builds on research by Maercker et al^46^ who found that adjustment processes in old age may benefit from personal experience of traumas and hardships during one’s life. It is possible that previous life experiences created a foundation of resilience within the individual to adapt to newer conflict, such as social isolation. Gallagher et al^47^ indicated that optimism and mastery after other sources of trauma such as surviving cancer, promote resilience. Additionally, it has been argued that some participants have certain personality traits that allow for more resilience.^18^ Going forwards, it may be possible to help identify community-dwelling older adults who may be at most risk of declining resilience based on their individual traits, community context and historical experiences throughout COVID-19.

Despite their resilience, some participants described pronounced impacts on their well-being as a result of their prolonged experience with mandated physical distancing measures. Participants described living in chronic fear, feeling despondent, and losing motivation for self-care. Other participants were more matter of fact in how they handled the restrictions, setting conscious intentions to remain optimistic and viewing the restrictions as a necessary action to keep them safe throughout the COVID-19 pandemic. Interestingly some participants who experienced fear and intense emotional impacts related to COVID-19 measures took concrete actions to shield and protect themselves from negative health impacts. They actively avoided the undue exposure to negative media influences and sought out social engagement with their immediate neighbors to replace lost in-person activities. Participants described how they personally distanced themselves from discussions involving COVID as a means to avoid conflicting engagements over political views and other distress arising as a result of government mandates. Key in this finding was that participants still had negative experiences, but they were able to respond in a way that fostered their resilience. This supports findings reported early in the pandemic that suggest that positive mindset and remaining busy and engaged were successful strategies to establish and foster emotional resilience.^3,25^ Conscious actions to help influence well-being have also been documented in COVID-19 research although in a different context. Older adults in the United Kingdom who used the internet searches primarily for the purpose of locating COVID-related health information experienced more pronounced feelings of loneliness than those who used it for social connection purposes.^48^ While older adults in the current study experienced varying impacts of the COVID-19 related social isolation and physical distancing mandates, those who fared best engaged in actions that provide examples of how their personal resources, (ie, personality) as well as environmental factors (ie, resilience of the community) shaped their ability to cope over a prolonged period of time.

Another key finding was that participants in this study leveraged the time they had as a result of COVID-19 constraints and lock down measures, to develop new skills or reconnect with previous skills they had learned in the past that had fostered their resilience, for example, learning a new language. This was similar to Xie et al^49^ finding of older adults engaging in new or previous hobbies to aid in adjustment at the beginning of the pandemic. Older adults in Mexico used smart technology to engage in new hobbies, such as learning new languages and gardening techniques and to continue with previous hobbies, such as knitting, meditating, and cooking recipes.^50,51^ The development of new skills to support the participants’ resilience is also consistent with one of Harden et al’s^3^ suggestions for possible interventions for decreasing social isolation at the individual level. Our findings extend the literature by suggesting that older adults continued to draw on pervious experiences and skills over a prolonged period of time throughout the pandemic. Furthermore, these findings suggest that helping older adults identify strengths and skills can be used as a means to foster their resilience across a range of situations and contexts.

## Implications and Limitations

Implications and next steps arising from these findings include recognition of the value and need to provide community initiatives that address aspects of equity, diversity and inclusion. These community initiatives can provide older adults with access to the skills, equipment, training, and supports necessary to continue to be engaged in their communities even if they are physically unable to leave their homes. As recent research by Strutt et al^52^ suggests, older adults who are comfortable in using technology will continue volunteering and work on committees even if managing physical constraints on in-person engagement. Moreover, Rivera-Torres et al^50^ posited that access to information and modern technology devices for leisure purposes benefited older adults’ mental health by reducing their risk of social isolation.

Of particular note, study findings suggest that in some cases the COVID-19 mandated restrictions on social gathering meant that older adults were able to realize unexpected benefits through being “gifted with time.” They were able to pause, re-evaluate how they spent their time and engage in activities like reading or learning new skills such as languages or yoga which they had little time for prior to COVID-19. The construct of social distancing-induced deceleration and its potential associated benefits such as reduced stress and improved sleep has been noted in other COVID-19 research.^53^ While not a widespread phenomenon, it highlights the importance of finding ways to help older adults find positive opportunities to grow from change. Community-based programs that focus on skill development as well as bringing people together using virtual connectivity could be particularly beneficial in such instances.

Practical implications for this research could include development of programs to increase digital literacy in older adults to support social connectivity as a contributor to older adults’ resilience against social isolation. Practitioners could provide programming on how to use the internet for online shopping, online banking, and video conferencing for those less comfortable with technology. Working to partner older and younger adults as a means to assist the older adults with technology proficiency could also serve to help young adults learn skills relating to resilience through the sharing of life experiences. Additionally, practitioners could create pamphlets of workshops and resources in the community where older adults can meet others or find services to meet their needs. Future research using longitudinal methods is needed to explore how older adults developed resilience against social isolation in the long term. Additionally, future research could also explore how to support older adults to develop skills to continue to create social connections in their community. It could be beneficial to develop screening tools that could help inform which segments of the community-dwelling older adult population could be most at risk of declining resilience during future public health emergencies or natural disasters.

Strengths of this study include a rich data set on the experiences of community-based older adults with COVID-19 related restrictions on in-person engagement. The findings of this research add to the knowledge base of factors that impact resilience in older adults who experience adversity. The study also has limitations. Including only English speaking participants excluded segments of the population. The sample likely overrepresents older adults who have access to technology and resources to meet their basic needs due to the nature of the methods required to conduct this study to meet social distancing requirements. In addition, the use of a research database to recruit participants suggests that those who consented to the study may not be representative of all older adults living in community settings. Additionally, the convenience sample is regionally limited, and lacking in gender, racial and socioeconomic diversity. The overrepresentation of the female gender could suggest that they were more comfortable in sharing personal experiences. However,| this would need to be investigated in another study dedicated to exploring gender and other differences which may be intersecting with one another and impacting their experiences. Future research should consider using targeted recruiting strategies and co-design approaches to help engage older adults that are more representative and inclusive of varying genders, ethnicities, cultures, and diverse groups within the broader population.

## Conclusion

In sum, the current study focused on how older adults reported on what they perceived helped foster resilience to prevent against social isolation during the COVID-19 pandemic. Some participants were open to learning new opportunities that enabled them to connect in the context of COVID-19 restrictions, but others were resistant to use technology for social engagement and conducting activities of daily living, such as online shopping. Although government policies have caused strain on participants by restricting their freedoms resulting in feelings of anxiety, loneliness, and depression, this was not the case for all participants. The participants who showed a willingness to learn, and a readiness to explore new ways to connect demonstrated their resilience by continuing to bounce back in the face of adversity even over a prolonged period of time. Going forwards, it will be important to develop strategies learned from the resilience demonstrated by these older adults so that those at risk of declining resilience can be supported in other pandemic or related situations.
